# The *vanR_Cd_* Mutation 343A>G, Resulting in a Thr115Ala Substitution, Is Associated with an Elevated Minimum Inhibitory Concentration (MIC) of Vancomycin in Clostridioides difficile Clinical Isolates from Florida

**DOI:** 10.1128/spectrum.03777-22

**Published:** 2023-05-01

**Authors:** Ishani Wickramage, Zhong Peng, Soumyadeep Chakraborty, Céline Harmanus, Ed J. Kuijper, Sally Alrabaa, Wiep Klaas Smits, Xingmin Sun

**Affiliations:** a Department of Molecular Medicine, Morsani College of Medicine, University of South Florida, Tampa, Florida, USA; b Department of Medical Microbiology, Leiden University Medical Center, Leiden, The Netherlands; c National Institute for Public Health and the Environment (RIVM), Bilthoven, The Netherlands; d Department of Internal Medicine, Morsani College of Medicine, University of South Florida, Tampa, Florida, USA; Tainan Hospital, Ministry of Health and Welfare

**Keywords:** *Clostridioides difficile*, antibiotic resistance, vancomycin, novel resistance-determining mechanisms, reduced susceptibility to vancomycin, single base pair mutation, van operon, vanGCd operon, vanRCd

## Abstract

Clostridioides difficile, the primary cause of nosocomial antibiotic-associated diarrhea, has a complex relationship with antibiotics. While the use of broad-spectrum antibiotics disrupts the gut microbiota and increases the risk of C. difficile infection (CDI), antibiotics are also the primary treatment for CDI. However, only a few antibiotics, including vancomycin, fidaxomicin, and rifaximin, are effective against CDI, and resistance to these antibiotics has emerged recently. In this study, we report the identification of two RT027 C. difficile clinical isolates (TGH35 and TGH64) obtained from symptomatic CDI-diagnosed patients in Tampa, Florida in 2016. These two strains showed an elevated minimum inhibitory concentration (MIC) of vancomycin (MIC = 4 μg/mL, compared to the EUCAST breakpoint of 2 μg/mL) and contained a *vanR_Cd_* 343A>G mutation resulting in a Thr115Ala substitution in the VanR_Cd_ response regulator. This mutation was absent in the vancomycin-sensitive control epidemic strain RT027/R20291. TGH64 was also resistant to rifaximin (MIC ≥ 128 μg/mL) and carried the previously reported Arg505Lys and Ile548Met mutations in RpoB. Furthermore, we report on the antimicrobial resistance (AMR) and genomic characterization of additional C. difficile isolates, including RT106/TGH120, RT017/TGH33, and RT017/TGH51, obtained from the same patient sample cohort representing the highly prevalent and regionally distributed C. difficile ribotypes worldwide. Considering that the VanR_Cd_ Thr115Ala mutation was also independently reported in seven C. difficile clinical isolates from Texas and Israel in 2019, we recommend epidemiological surveillance to better understand the impact of this mutation on vancomycin resistance.

**IMPORTANCE** The perpetually evolving antimicrobial resistance (AMR) of C. difficile is an important contributor to its epidemiology and is a grave concern to global public health. This exacerbates the challenge of treating the infections caused by this multidrug-resistant causative organism of potentially life-threatening diarrhea. Further, the novel resistance-determining factors can be transferred between different strains and species of bacteria and cause the spread of AMR in clinical, environmental, and community settings. In this study, we have identified a mutation (*vanR_Cd_* 343A>G) that causes a Thr115Ala substitution and is linked to an increased MIC of vancomycin in clinical isolates of C. difficile obtained from Florida in 2016. Understanding the mechanisms of AMR, especially those of newly evolving strains, is essential to effectively guide antibiotic stewardship policies to combat antibiotic resistance as well as to discover novel therapeutic targets.

## OBSERVATION

Clostridioides difficile, which is a cause of mild to life-threatening diarrhea that was responsible for 12,800 deaths in the USA in 2017, has been declared by the CDC as one of the top five urgent antibiotic-resistant threats (1). The emergence of new strains, which are often more virulent and antibiotic-resistant, has been associated with the recent increase in the prevalence and severity of CDI (2).

As a life-threatening disease with a high incidence of recurrence and limited effective therapeutic options, the resistance of C. difficile to vancomycin, which is the first line of therapy that is recommended for CDI treatment, is a grave public health concern (2, 3). Vancomycin inhibits bacterial cell wall synthesis by binding with high affinity to the d-Ala-d-Ala C terminus of peptidoglycan precursors, which thereby prevents the addition of late precursors to the nascent peptidoglycan chain (4). A gene cluster called the “*van* operon” has been described to mediate vancomycin resistance in enterococci (4). The two-component regulatory system of *van* operons encompasses a sensor histidine kinase (VanS) and a response regulator (VanR). The *vanG*-type of operon contains the resistance genes VanG (d-Ala-d-Ser ligase), VanXY (a bifunctional D,d-dipeptidase/d,d-carboxypeptidase), and VanT (serine racemase). When vancomycin is sensed by the membrane-bound VanS, it undergoes the ATP-dependent autophosphorylation of a histidine residue. This phosphoryl group is then transferred to the cytoplasmic VanR, which, in turn, transcriptionally activates the expression of downstream resistance genes. VanT converts l-Ser to d-Ser and VanG ligates d-Ala and d-Ser, forming low-affinity precursors and modifying the vancomycin binding target. VanXY hydrolyzes peptidoglycan precursors that end with d-Ala residues, thereby eliminating the high-affinity binding targets of vancomycin (4). However, although a functional *vanG* operon-like gene cluster called “*vanG_Cd_*” has been found in about 85% of C. difficile clinical isolates, it was not associated with vancomycin resistance in C. difficile (5).

We previously reported 139 C. difficile clinical isolates that were obtained from symptomatic patients who were diagnosed with CDI in Tampa, FL, USA (6). Based on broth microdilution-based screening for antimicrobial susceptibility, we selected isolates that showed a reduced susceptibility to multiple antibiotics and conducted capillary PCR ribotyping at the Dutch National Reference Laboratory at the Leiden University Medical Center (LUMC), using a standardized protocol (7). Considering that the epidemiology of C. difficile shows distinct geographical distributions, we selected five isolates to represent the most prevalent C. difficile ribotypes that are found in different geographical locations around the world, namely, RT027 (Europe and North America) (2), RT106 (USA) (8), and RT017 (Asia) (9), for further analysis in the present study.

**FIG 1:**
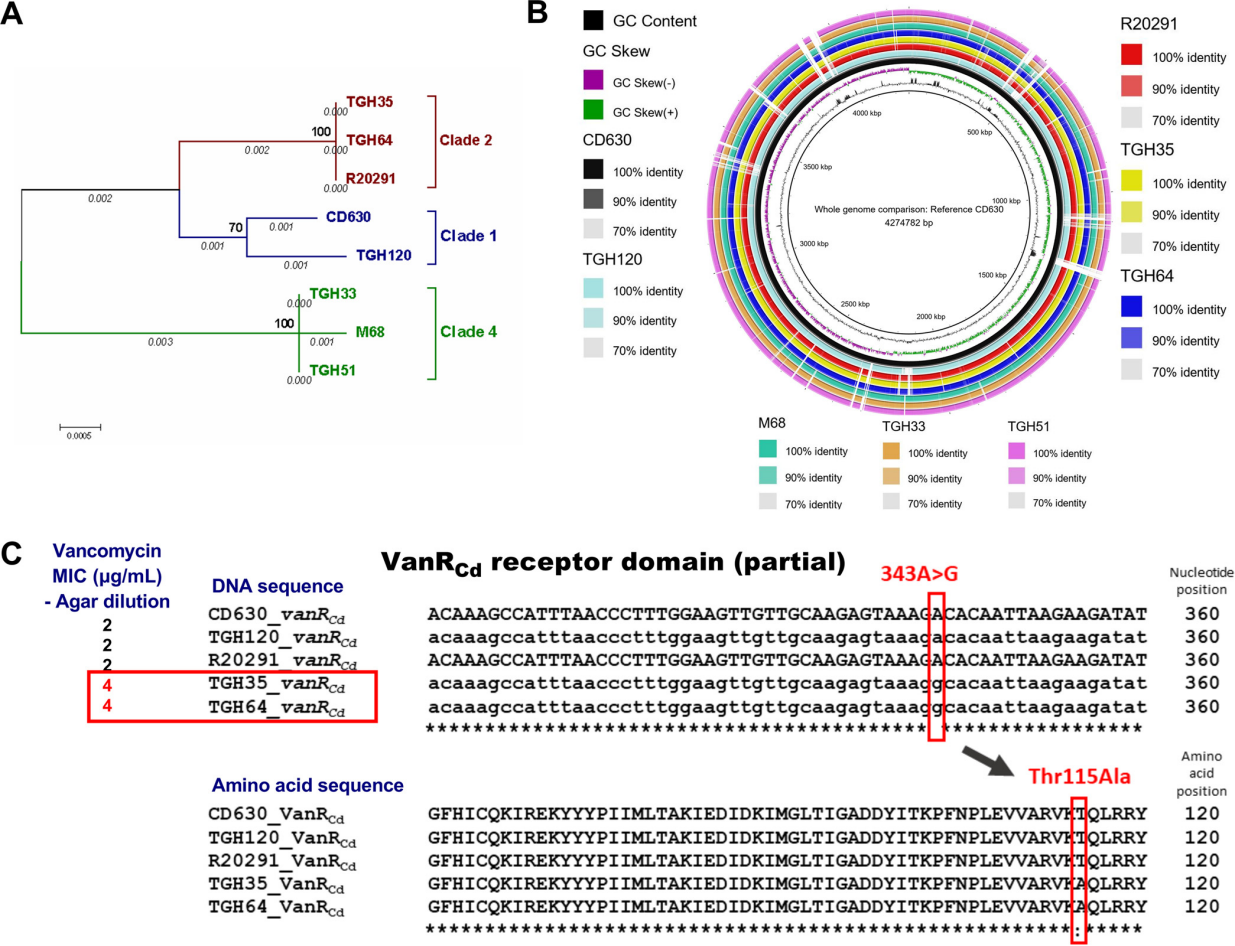
Genome analysis of the five C. difficile isolates and three control strains CD630, R20291, and M68 (GenBank accessions CP010905.2, FN545816.1, and FN668375.1, respectively). (A) Phylogenetic tree generated with seven housekeeping genes, using MEGA7 software. The seven housekeeping genes were concatenated to generate a supergene alignment using MUSCLE, and a phylogenetic tree was generated using the maximum likelihood method. A general time reversible (GTR) model was used to generate the substitution model using gamma distributed with invariant sites (G+I) and 1,000 bootstrap replications. (B) BLAST ring image generator-based comparison and visualization of the whole-genomes. (C) Multiple sequence alignment (Clustal Omega) of the *vanR_Cd_* gene (upper panel) and amino acid sequence (lower panel) in comparison with the vancomycin MICs obtained via the agar dilution method (left) of the novel isolates and control strains. The *vanG_Cd_* operon is absent in the RT017 strains M68, TGH33, and TGH51.

We cultured the five selected C. difficile isolates, namely, TGH35, TGH64, TGH120, TGH33, and TGH51, in brain heart infusion (BHI) broth (Sigma) at 37°C under anerobic conditions. Following genomic DNA extraction with a kit (Qiagen) and whole-genome sequencing using paired-end libraries and an Illumina HiSeq 3000 platform, we *de novo* assembled reads into contigs using Qiagen CLC Genomics Workbench 11.0.1 (10) (Table S1). After ordering the contigs against the reference CD630 genome (GenBank accession: CP010905.2) using Mauve (v2.4.0) (11) and annotating them using Rapid Annotations using Subsystems Technology (RAST) (12), we compared the whole-genomes of the five isolates with three reference strain genomes, namely, CD630/RT012/Clade1, R20291/RT027/Clade2, and M68/RT017/Clade4 (GenBank accessions: CP010905.2, FN545816.1, and FN668375.1, respectively), and we visualized them using the BLAST Ring Image Generator (BRIG) (13). The housekeeping genes *adk*, *atpA*, *glyA*, *sodA*, *dxr*, *recA*, and *tpi* were used for web-based multilocus sequence typing (MLST) on a CGE MLST platform (14), and a phylogenetic analysis was conducted using MEGA7 (15). Prophages were predicted using the PHAge Search Tool (PHAST) (16). We searched for plasmids using CGEPlasmidFinder-2.0 (17) and manually evaluated the presence of sequences corresponding to pCD-METRO (GenBank: OM972905.1) (18). We used the comprehensive antibiotic resistance database (CARD) to search for putative AMR genes using the Resistance Gene Identifier (RGI) tool for resistome predictions, based on homology and SNP models (19). The features of the characterized genomes are illustrated in Fig. 1, Table 1, and Table S2.

**TABLE 1:** Genome features, MICs by agar dilution, AMR determinants, and predicted intact prophages of the isolates^*a*^

Strain	Ribotype	MLST^11^	Clade	MIC (μg/mL) determined by agar dilution (sensitivity criteria, source)				AMR determinants (CARD database)					Predicted intact prophages (PHAST)
				VAN (S ≤ 2, R > 2; EUCAST ECOFFs)	MTZ (S ≤ 8, I = 16, R ≥ 32; CLSI)	FDX (S < 1, I > 1; literature)	RFX (S ≤ 0.0.004, I = 0.004 to 16, R ≥ 16; literature)	ARO term	AMR gene family	Resistance mechanism	% identity of matching region	% length of reference sequence	
TGH35	RT027	ST1	2	4	0.5	≤0.5	≤0.125	APH(3′)-Ia	APH(3′)	Antibiotic inactivation	100.0	30.63	PHAGE_ Clostr_ phiMMP01_ NC_028883
								cdeA	Multidrug and toxic compound extrusion (MATE) transporter	Antibiotic efflux	99.09	100.00	
								APH(3′)-Ia	APH(3′)	Antibiotic inactivation	96.77	67.90	
								vanRG	Glycopeptide resistance gene cluster, vanR	Antibiotic target alteration	77.45	99.15	
								tet(W/N/W)	Tetracycline-resistant ribosomal protection protein	Antibiotic target protection	69.28	100.00	
								vanXYG	Glycopeptide resistance gene cluster, vanXY	Antibiotic target alteration	58.82	105.51	
TGH64	RT027	ST1	2	4	0.25	≤0.5	≥128	dfrF	Trimethoprim resistant dihydrofolate reductase dfr	Antibiotic target replacement	100.0	100.00	PHAGE_ Clostr_ phiMMP01_ NC_028883
								cdeA	MATE transporter	Antibiotic efflux	99.32	100.00	
								vanRG	Glycopeptide resistance gene cluster, vanR	Antibiotic target alteration	77.45	99.15	
								vanXYG	Glycopeptide resistance gene cluster, vanXY	Antibiotic target alteration	58.82	105.51	
								tet(W/N/W)	Tetracycline-resistant ribosomal protection protein	Antibiotic target protection	69.28	100.00	
								ermB	Erm 23S ribosomal RNA methyltransferase	Antibiotic target alteration	98.78	98.79	
TGH120	RT106	ST42	1	2	0.5	≤0.5	≤0.125	APH(3′)-Ia	APH(3′)	Antibiotic inactivation	100.0	29.89	-
								cdeA	MATE transporter	Antibiotic efflux	99.32	100.00	
								vanRG	Glycopeptide resistance gene cluster, vanR	Antibiotic target alteration	77.87	99.15	
								vanXYG	Glycopeptide resistance gene cluster, vanXY	Antibiotic target alteration	58.82	105.51	
TGH33	RT017	ST37	4	2	0.5	≤0.5	≤0.125	cdeA	MATE transporter	Antibiotic efflux	98.41	100.00	PHAGE_ Clostr_ phiCD506_ NC_028838 PHAGE_ Clostr_ phiMMP01_ NC_028883
								APH(3′)-Ia	APH(3′)	Antibiotic inactivation	99.1	40.96	
								APH(3′)-Ia	APH(3′)	Antibiotic inactivation	100.0	28.7	
TGH51	RT017	ST37	4	1	0.5	≤0.5	≤0.125	cdeA	MATE transporter	Antibiotic efflux	98.41	100.00	PHAGE_ Clostr_ phiCD506_ NC_028838 PHAGE_ Clostr_ phiCDHM19_ NC_028996 PHAGE_ Clostr_ phiCDHM19_ NC_028996 PHAGE_ Clostr_ phiMMP01_ NC_028883
								APH(3′)-Ia	APH(3′)	Antibiotic inactivation	97.79	99.26	

a The MICs of vancomycin via the agar dilution method for both control strains RT027/R20291 and RT012/CD630 were 2 μg/mL. The MICs of vancomycin were further determined via BHI broth microdilution: TGH35 (2 μg/mL), TGH64 (4 μg/mL), TGH120 (1 μg/mL), TGH33 (1 μg/mL), TGH51 (1 μg/mL), and R20291 (1 μg/mL). The MICs of vancomycin were also tested via Brucella blood agar dilution and E-tests at LUMC: TGH35 (1 μg/mL), TGH64 (2 μg/mL), TGH120 (0.25 μg/mL), TGH33 (0.5 μg/mL), R20291 (0.5 μg/mL). An EMBL Clustal Omega-based multiple sequence alignment was performed to explore the presence of the mutations that were previously reported to be associated with a reduced susceptibility to vancomycin in *in vitro* C. difficile strains. Pro108Leu in MurG was detected in all of the isolates in the present study, and G733T in *rpoC* was detected in none of them. MIC, minimum inhibitory concentration; AMR, antimicrobial resistance; ECOFFs, epidemiological cutoff values; VAN, vancomycin; MTZ, metronidazole; FDX, fidaxomicin; RFX, rifaximin; MLST, multi locus sequence typing; CARD, comprehensive antibiotic resistance database; ARO, Antibiotic resistance ontology. Resistance Gene Identifier criteria used by the CARD database: Strict. Detection criteria: Protein homolog model.

The antibiotic susceptibility testing (AST) of the isolates to the currently clinically used antibiotics for CDI treatment, namely, vancomycin, metronidazole, fidaxomicin, and rifaximin, was conducted using the reference standard agar dilution method, based on the Clinical and Laboratory Standards Institute (CLSI) guidelines M11-A7 (volume 27, no 2, ISBN:1-56238-626-3), using the epidemic RT027/R20291 C. difficile strain as a control with a minimum of three technical replicates in two independent experiments. While all strains were susceptible to metronidazole and fidaxomicin, high resistance to rifaximin (MIC ≥ 128 μg/mL) was detected only in TGH64. Rifaximin, which is an adjunct therapeutic for CDI, acts by binding to the β-subunit of RNA polymerase (RpoB), which thereby inhibits bacterial RNA synthesis (20). Since mutations in the rifamycin resistance-determining region (RRID) of RpoB have been previously associated with rifamycin resistance in C. difficile without imposing a fitness cost (20), we performed a EMBL Clustal Omega-based multiple sequence alignment (MSA) (21) on the *rpoB* genes in all isolates. We detected two *rpoB* SNPs in TGH64, namely, 1514G>A and 1644A>G, that result in Arg505Lys and Ile548Met, respectively. This combination of mutations has previously been associated with rifamycin resistance in C. difficile clinical isolates. Structural modeling by Dang et al. suggested that Arg505Lys results in the loss of the energetically favorable pi-stacking interactions between RRID and rifaximin, which inhibits drug binding, thereby leading to resistance (20).

Two isolates that were ribotyped as RT027, namely, TGH35 and TGH64, showed elevated vancomycin MIC (MIC = 4 μg/mL; compared to the EUCAST breakpoint of 2 μg/mL) (Table 1) values. We further assessed the vancomycin susceptibility of these strains via two other methods: the BHI broth microdilution, based on the CLSI guidelines, and Etest, which was conducted at LUMC. In agreement with the results of previous studies, both of these methods gave lower MIC values than were obtained via the reference agar dilution method for several strains (22, 23). However, with all three methods, the elevated vancomycin MIC in TGH35 and TGH64 persisted compared to the other strains.

CARD-based AMR prediction revealed two genes of the *vanG_Cd_* operon, namely, *vanR_Cd_* and *vanXY_Cd_*, in TGH35, TGH64, and TGH120. While *vanXY_Cd_* was identical in all three isolates, the *vanR_Cd_* of TGH120 differed from the two RT027 strains (Table 1). Therefore, we performed a MSA of the entire *vanG_Cd_* operon in these isolates with the reference strains CD630 and R20291. The *vanG_Cd_* operon is absent in the TGH33, TGH51, and M68 strains. Remarkably, only one nucleotide difference was detected in the whole >6 kb *vanG_Cd_* operon comparison between the five strains: 343A>G in the *vanR_Cd_* gene of TGH35 and TGH64 (Fig. 1C). The resulting sense mutation Thr115Ala in the receptor domain of VanR_Cd_ likely affects the expression of the downstream resistance genes. We reported this mutation at the ASM Microbe Annual Conference in June of 2019 (24). Later, the VanR_Cd_ Thr115Ala mutation was also independently reported in seven C. difficile clinical isolates from the Texas Medical Center, USA as well as in two C. difficile clinical isolates from Israel (25). These isolates showed the constitutive expression of the *vanG_Cd_* operon and elevated MICs of vancomycin, which could be reversed in the VanR_Cd_-mutant isolates by silencing *vanG_Cd_*, whereas *vanG_Cd_* silencing had no effect on the MIC of the control R20291 strain. Shen et al. also used the structural homology modeling of VanR_Cd_ to propose that the Thr115Ala substitution provides better stability for its interaction with DNA, thereby enhancing the capability for the transcriptional activation of downstream genes. Since single base pair mutations under selection can quickly lead to the development of resistance, the present work highlights the need for epidemiological surveillance to monitor the prevalence of this mutation, especially in vancomycin-treated patients, to better understand its effects on the resistance to an antibiotic that is currently crucial in the treatment of C. difficile infection.

### Data availability.

The whole-genome shotgun projects for the strains TGH35, TGH64, TGH120, TGH33, and TGH51 have been deposited into DDBJ/ENA/GenBank under the accession numbers JAJNGZ000000000, JAJNHA000000000, JAJNHB000000000, JAJNHC000000000, and JAJNHD000000000, respectively.
