# Supplementary material for: The vanRCd Mutation 343A>G, Resulting in a Thr115Ala Substitution, Is Associated with an Elevated Minimum Inhibitory Concentration (MIC) of Vancomycin in Clostridioides difficile Clinical Isolates from Florida
Source: Microbiol Spectr. 2023 May 1;11(3):e03777-22. doi: 10.1128/spectrum.03777-22 (PMC10269549; doi:10.1128/spectrum.03777-22)
Supplement: Supplemental file 1 — Supplemental material. Download spectrum.03777-22-s0001.pdf, PDF file, 0.05 MB [file spectrum.03777-22-s0001.pdf]

TABLE S1 Genome assembly parameters

Contig measurements	TGH35	TGH64	TGH120	TGH33	TGH51
N75	80573	80573	104765	135548	125864
N50	141174	150740	490002	218943	205835
N25	236835	236932	604901	377544	373192
Minimum	1019	1019	1033	1004	1029
Maximum	353588	353716	998276	489189	489190
Average	72423	70165	108667	86928	78895
Count	57	59	37	48	54
Total	4128136	4139722	4020692	4172520	4260308

These genome assemblies were generated with CLC Genomics Workbench 11.0.1 (Qiagen) with the following parameters: Word size 64, Minimum contig length 1000, Mismatch cost 2, No scaffolding.

TABLE S2 Genomic features of the novel isolates

Strain	Genome size (bp)	GC%	CDS	Full length toxin-encoding genes
TGH35	4128136	28.61	3798	*tcdA*, *tcdB*, *cdtA*, *cdtB*
TGH64	4139722	28.59	3832	*tcdA*, *tcdB*, *cdtA*, *cdtB*
TGH120	4020692	28.33	3636	*tcdA*, *tcdB*
TGH33	4172520	28.67	3827	*tcdB*
TGH51	4260308	28.5	3989	*tcdB*

Rapid Annotation using Subsystems Technology (RAST) webserver was used to annotate the genomes. CDS: coding sequences.
